# Information consumption and firm size

**DOI:** 10.1098/rsos.240027

**Published:** 2024-11-06

**Authors:** Edward D. Lee, Alan P. Kwan, Rudolf Hanel, Anjali Bhatt, Frank Neffke

**Affiliations:** ^1^ Complexity Science Hub, Vienna, Austria; ^2^ Hong Kong University, Hong Kong, People's Republic of China; ^3^ Harvard Business School, Boston, MA, USA

**Keywords:** reading, firms, scaling, information

## Abstract

Social and biological collectives exchange information through internal networks to function. Less studied is the quantity and variety of information transmitted. We characterize the information flow into organizations, primarily business firms. We measure online reading using a large dataset of articles accessed by employees across millions of firms. We measure and relate quantitatively three aspects: reading volume, variety and firm size. We compare volume with size, showing that firm sizes grow sublinearly with reading volume. This is like an economy of scale in information consumption that exaggerates the classic Zipf’s law inequality for firm economics. We connect variety and volume to show that reading variety is limited. Firms above a threshold size read repetitively, consistent with the onset of a coordination problem between teams of employees in a simple model. Finally, we relate reading variety to size. The relationship is consistent with large firms that accumulate interests as they grow. We argue that this reflects structural constraints. Taking the scaling relations as a baseline, we show that excess reading is strongly correlated with returns and valuations. The results indicate how information consumption reflects internal structure, beyond individual employees, as is likewise important for collective information processing in other systems.

## 1. Introduction

Information exchange facilitates collective behaviour including coordination in schools of fishes or flocks of birds [1–4], conflict levels in primate society [5], team performance [6] and organizational function [7,8]. In each of these cases, the transmission of information is structured by cognitive constraints [9], social strategy and relationships [10] or simply distance [11]. One particularly compelling example is the business firm. Individuals throughout the firm hierarchy acquire and process information to make decisions and retransmit information to different parts of the organization [12,13]. Importantly, social structures such as bridges or weak ties [14,15] mediate the flow of information, a perspective that has inspired the statistical physics [16,17] and computational social science [18] of social networks. Pioneering work has gone into characterizing the structural properties of the networks. In the field of collective knowledge, the properties of nodes include tacit knowledge, worker capabilities and social skills [19]. Edges represent co-worker complementarities [20]. Structural properties may reflect organization and hierarchy [21]. On the other end, others focus on the output of the computation done by the networks such as how patents reflect combinatorial innovation [22–24]. In a related vein, the management literature has investigated how firms absorb [13], process and use information through investment in R&D and internal communications [8]. To connect the internal structure to a firm’s output, we need to determine the dynamics of information flow. Yet, information flow and its contents are largely unseen in business firms. Here, we are able to peer into one part of it: online reading. By relating scaling patterns in the data to one another, we take a step towards quantitatively modelling the volume and variety of information in organizations. We establish quantitative measures that can help connect the scaling to principles that mediate or shape collective behaviour more broadly.

We look into the prodigious information consumption of millions of organizations globally, the vast majority of which are business firms. To do so, we analyse an extensive dataset of ‘intent data’ aimed at gauging customer interest [25,26]. The data consists of hundreds of millions of records of content accessed by firm employees within a large universe of publishers including household names in financial and general news media along with more specialized sites. They span technology, marketing, legal, biotech, manufacturing and a wide range of business services [25]. We focus on a two-week period between the dates of 10 June and 23 June, 2018, which we expect is generally representative of the dataset as we detail further in the electronic supplementary material, appendix A. The limited time window also ensures that proprietary preprocessing steps used to generate the data remain consistent. In principle, the data would allow us to determine which news article an anonymized employee at a firm accessed and when. For each article, we have up to 10 associated topics that have been identified with a proprietary topic modelling algorithm (more details in Kwan & Zhu [25]). These different properties permit us to analyse employee reading at different scales of resolution from the individual articles, which then belong to sets of content pages or ‘sources’, and that may overlap in broader ‘topics’ as diagrammed in the electronic supplementary material, figure S1 [27,28]. Importantly, the comprehensive nature of the dataset allows us to obtain a multiscale portrait of how firms seek out information down to the individual acts of information acquisition.

We focus on large-scale, population-wide aspects and establish trends between the volume of information consumption, its variety and measures of firm size. In the first part of the paper, we relate reading volume with firm size. We find that firm size scales sublinearly with volume, leading to an inequality of information consumption that exaggerates the Zipf’s law inequality in firm size [29]. By contrast, some measures of variety scale linearly. This suggests that reading volume reflects a different organizational structure than that reflected in economic metrics of size. Then, we relate volume with variety to find that large firms tend to be redundant readers. We relate the limited variety of reading to coordination limits in firms, and we propose that the scaling of teams of employees is a crucial part of predicting how diversely large firms read. Finally, by exploring the relationship between variety and firm size, we predict two qualitative extremes of firm reading strategies, where either firms’ asset-to-topic ratio increases or the ratio decreases. The model predicts that nearly all firms tend to the former, which aligns with conventional wisdom for how firms focus on core competencies [30,31]. Interestingly, the increasing concentration of assets per topic is in contrast with the economy of scale for reading volume or elementary bits of information, suggesting that the way that firms use information at the scale of employees is fundamentally different from its use at the scale of the organization. This echoes our main finding that reading behaviour displays quantitative traces of organizational structure and thus delivers insight on the firm as an information processing machine.

## 2. Economy of scale in information?

**Figure 1.**
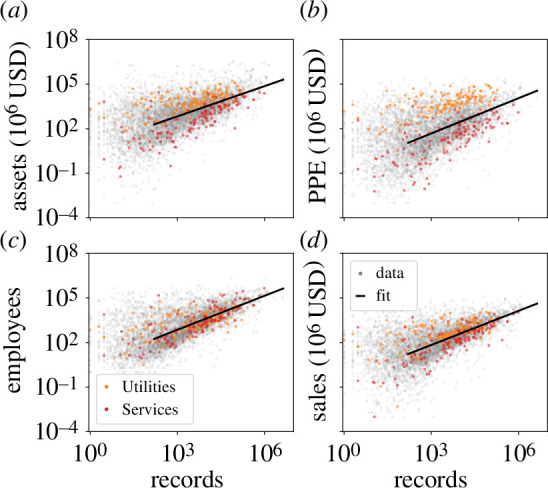
Scaling of firm size measures (*a*) assets, (*b*) plants, property, equipment (PPE), (*c*) employees, and (*d*) sales with record count. North American Industrial Classification System sectors utilities (orange), professional, scientific and technical services (red), and all other (grey) firms. Black line shows a power law fit to equation 2.1 with exponents (*a*) β=0.73±0.01 , (*b*) β=0.82±0.02 , (*c*) β=0.79±0.01 and (*d*) β=0.80±0.01 using one s.d. from bootstrapped fits as error bars. Fitting range R≥160 given by the fit to the distribution in figure 2*e*.

As a first step to characterizing how firms seek out information, we consider the relationship between the volume of information acquisition, the number of times a firm is recorded in the database, or ‘records’, and conventional measures of firm capital. In figure 1, we plot for public firms in the COMPUSTAT database the value of firm assets, plants, property, equipment (PPE), the number of employees and sales against the number of records. Building on previous work on scaling in firms [32], we consider a power law relationship between economic measure Y and records R :

**Figure 2.**
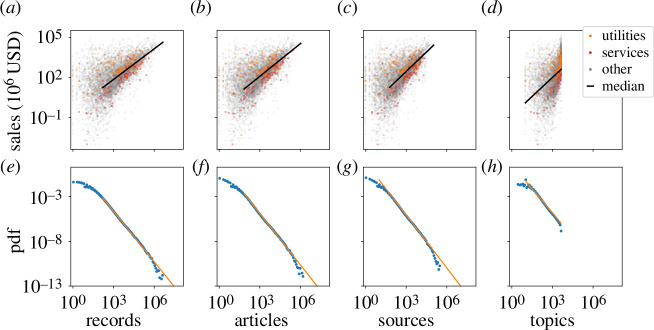
(*a−d*) Scaling of sales with information variables. Mining (green), service (red) and all other (grey) firms. Black line is the scaling fit. Scaling exponents are β=0.80±0.01 , β=0.85±0.01 , β=1.03±0.02 and β=1.16±0.03 using bootstrapped error bars. Fits are only to data points above the lower cut-off. Lower cut-off is given by the fit to the distributions in the panel directly below except for topics, where the lower cut-off is set *a priori* to the topic relevancy vector size of 10. (*e−h*) Distribution of the number of records α=1.88 , articles α=1.92 , sources α=1.97 and topics α=1.80 per firm shows power law scaling in the tails. A standard fitting procedure involving maximum likelihood for the exponent α with the Kolmogorov−Smirnov statistic for the lower bounds returns $x_{\text{min}}=160$ , $x_{\text{min}}=70$ and $x_{\text{min}}=62$ [33]. For topics, the lower bound is fixed at $x_{\text{min}}=10$ and the maximum at $x_{\max}=4338$ . Our simplified scaling model does not capture the curvature in (*d*). See the electronic supplementary material, table S2 for further exponents.


(2.1) $$Y=AR^{\beta },$$

for some positive constant A and positive exponent β . This is equivalent to a regression on a logarithmic scale, where the slope is β and the y -intercept logA when equation (2.1) is transformed to logY=βlogR+logA . Importantly, the exponent β is independent of the units of R and Y , the conversion between which is separately captured in A . We find that all economic measures scale sublinearly with the number of records with scaling exponents of about β≈3/4 (electronic supplementary material, table S2). Furthermore, it tends to be the case that a category of service firms (as indicated by the North American Industrial Classification System (NAICS), red points falling below the fit line) have more records per unit of economic measure as compared to firms in utilities (orange points falling above the fit line). They are not as clearly differentiated for employees, but the estimate of the number of employees in COMPUSTAT is known to be poor. While the services category includes a broad swathe of industries and thus displays wide variation along the vertical, the trend is consistent with the notion that firms in the service sector are on the whole more knowledge-intensive when compared to utilities.

A record, however, is a rather rudimentary measure of information flow. Within any given record, we are also given the article that was accessed, the page on which it was published or ‘source’, and the topics that were relevant to the article, the latter a derivative measure obtained from proprietary topic modelling (see electronic supplementary material, appendix A for more details). Since these constitute progressively larger groupings of individual records, we might anticipate the economic size to scale differently with the information measures at coarser granularity. As we show in figure 2, we find that sales grow faster with the number of articles ( β=0.85±0.01 ), with sources ( β=1.03±0.02 ) and with topics ( β=1.16±0.03 ), respectively, using a least-squares fit on logarithmic axes. The sublinearity with records indicates that the typical increase in elementary counts of information access grows faster than the increase in sales such that the effective cost of accessing *new* information decreases with reading volume or articles, but this is not the case for sources and topics.

Importantly, the scaling between economic and information measures is closely aligned with the heavy-tailed distributions of information search. This would mean that our results extracted for a small subset of public firms in COMPUSTAT are consistent with the distribution from the millions of firms in the reading data. We check this by using the observation that the distribution of firm receipts, here given by sales Y , follows a Zipf’s law such that $p(Y)\sim Y^{-2}$ [29]. Then, if it is the case that the distribution of information quantity X obeys $p(X)\sim X^{-\alpha }$ , we can use the transformation p(S)dS=p(X)dX and the scaling relation $Y=AR^{\beta }$ to obtain the exponent relationship:


(2.2) α=β+1.

Using the values of β found above, we obtain the predictions $\hat{\alpha }=1.80\pm 0.01$ , $\hat{\alpha }=1.85\pm 0.01$ , $\hat{\alpha }=2.03\pm 0.02$ and $\hat{\alpha }=2.16\pm 0.03$ .

Next, we fit the probability distributions by the number of records, articles and topics in figure 2 using a standard method [33], which gives us a direct estimate of α instead of from using the right hand side of equation (2.2). From this method, we obtain α=1.88±0.01 , α=1.92±0.00 , α=1.97±0.01 and α=1.80±0.00 for records, articles, sources and topics, respectively. We note that the fit to topics provides a negative check because it should fail. After all, the fit to topics is statistically distinguishable from a power law, is limited in its range, and the estimate of β from figure 2*d* does not account for the curvature in the plotted data. Accordingly, we find that the exponent scaling relationship in equation (2.2) is least well satisfied for topics with error of $\Delta \equiv \hat{\alpha }-\alpha =0.36\pm 0.03$ , but is better satisfied for the remaining quantities Δ=−0.08±0.01 , Δ=−0.07±0.01 and Δ=0.06±0.02 for records, articles and sources, respectively. While one has to be careful with start-ups and the smallest firms, which show much more variability in information and economic behaviour [32], our scaling models establish a basis for comparison relative to an expected trend. Indeed, that the exponents are close to satisfying the exponent relationship equation (2.2)—despite β having been extracted for a small subset of public firms in COMPUSTAT and α from all firms in the reading data—indicates that our scaling approximation is reasonably self-consistent.

The exponents α<2 for records and articles indicate heavy-tailed distributions and thus strong inequality in how firms read. By contrast, economic size distributions have an exponent α=2 . This means that along with elementary measures of information, but not the aggregated measures of sources or topics, we find that the largest firms have a disproportionately larger information footprint.

## 3. Limits to information variety

**Figure 3.**
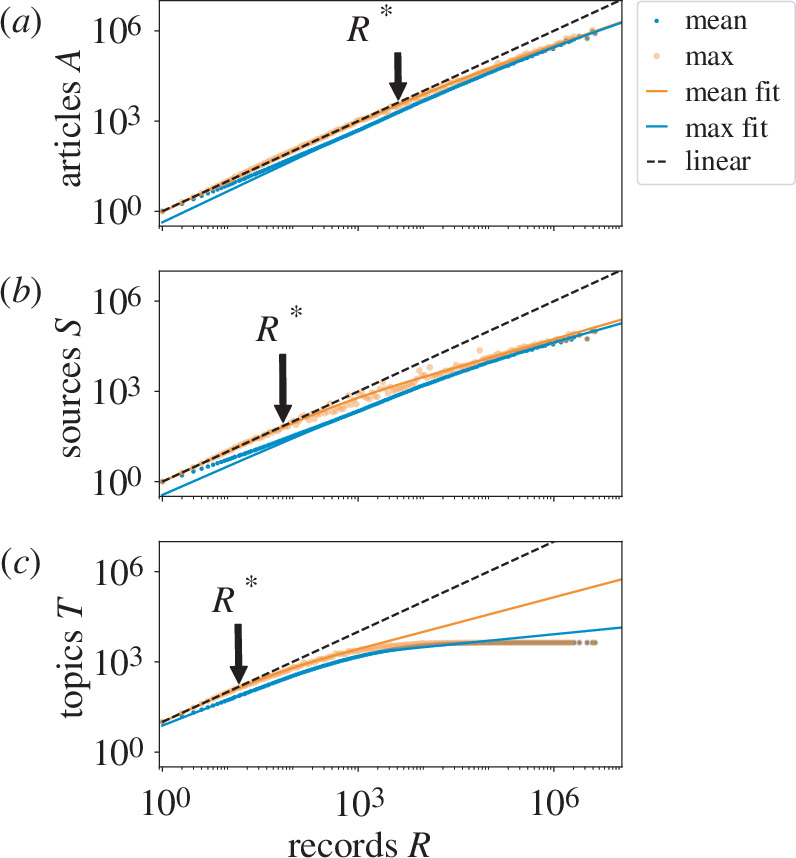
Diversity of information collected by firms as Heaps’ plots for firm reading. Growth of (*a*) articles A , (*b*) sources S , and (*c*) topics T with number of records R along with fits of information-overlap model as lines. For a given logarithmic bin R , we show the firms with maximal variety in reading (orange markers) and firms with mean reading variety (blue markers). While the total number of articles ( A~40 million) and sources (S ∼ 8 00 000) is much greater than what any single firm accesses in the subsample, the number of topics is bounded at T=4,338 . To avoid overfitting to the cut-off, we scan over a range of values and only take fit values that are of sufficiently low error and do not violate physical limits (more details in electronic supplementary material, appendix D). Estimated team size scaling exponents are b≈3/10 for both mean and max curves for articles. For sources, b≈1/3 and b≈2/5 for mean and max, respectively. For topics, b≈1/2 and b≈2/3 . Points $R^{*}$ at which the maximal curves fall below 90% of the 1 : 1 line are indicated with arrows R=1645 records for articles, R=41 records for sources and R=18 records for topics.

More reading does not necessarily imply new information for the firm. In the Heaps’ plot in figure 3, we compare the number of unique articles A , sources S or topics T with the total number of times the firm accessed content or left a record R [34]. For a given logarithmic bin for records R , we plot the average number of items read by firms (blue markers) below the maximum (orange). Both data points grow sublinearly with R in every panel. This is sensible because we know that a single employee might read the same article several times or share it with colleagues such that this plot would at maximum trace the black, dashed and one-to-one line. Furthermore, the three Heaps’ plots all show qualitatively similar patterns of two distinct regimes: the least read (small R ) firms saturate the variety of articles and the most read (large R ) firms fail to saturate this curve. This means that small firms with the most diverse reading tendencies saturate the maximum number of articles they can read per record but that at a larger size, the most diverse readers peel away from linear growth, indicating that the same articles A , sources S and topics T are reread.

**Figure 4.**
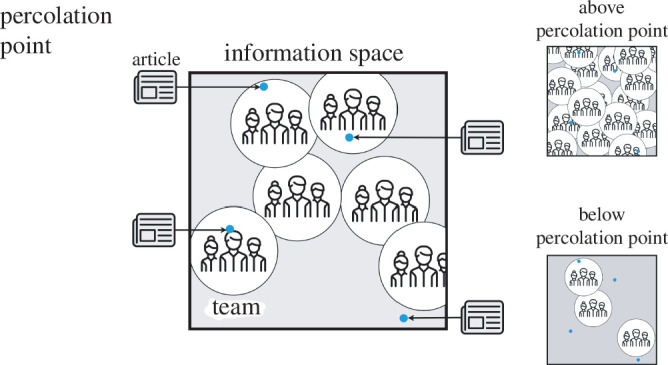
Diagram of information processing model. Firm consists of organizational units, each with a range of expertise in a high-dimensional space of information content, here projected onto two dimensions. If an article falls into the range of expertise of a unit, the firm can realize a benefit (equation 3.1). In small firms below the percolation point, teams hardly fill the space and in large firms above the percolation point teams overlap substantially. The percolation point is the point at which the majority of units begin to touch. Below it, organizational units generally do not overlap, and above it organizational units generally do. Our formulation is general and allows for a wide range of interpretations that are consistent with the probabilistic formulation such as limited variations of goal-directed search, random realization of the economic benefit, biased distribution of information and teams in the space, information that is passed around the firm to find an expert, among others.

We model how such a transition may occur with a simplified picture of how firms process information and extract benefits, as in figure 4. We picture each record to be a point in a high-dimensional information space as indicated by the arrows. Each employee has some expertise, corresponding to a volume in the same space; this can be represented as a ‘ball’ with a characteristic radius r . Then, we assume that firms extract some economic benefit B from each piece of information if there is overlap between the expertise of an employee and the information content of a record. The chance that the record intersects with the expertise of the employee is the ratio of the size of the ball to the volume of the space, or the fraction p . Considering a firm with N employees in a large information space, the probability that the piece intersects with the expertise of at least one employee is $1-{\left(1-p\right)}^{N}$ . The result is exponential convergence to full coverage with employee number, or the prediction of a sudden turning point as a firm goes from a small size with a sparse covering to a large one with a dense covering of the information space.

The level at which useful information is extracted, however, is typically not relegated to a single employee but an organizational unit (e.g. teams, branches, etc.), which may serve as a collection of expertise relatively independent of other units. Then, it is more accurate to consider a scaling relationship of the organizational unit with the number of employees $N^{b}$ for a positive and sublinear exponent b , which implies that the probability of finding at least one unit with the right expertise is $1-{\left(1-p\right)}^{cN^{b}}$ , for a positive constant c and where b=1 as considered above corresponds to a firm with no organizational grouping. Simultaneously, firms are limited in the total amount of incoming information they can process regardless of overlap with expertise, a quantity that we model as scaling with the number of employees of $N^{a}$ . As before, the typical number of successful intersections between an information piece and an organizational unit yields a total economic benefit B . Assuming that cost of new information is proportional to its frequency (i.e. it pays for itself),^1^ we would expect that B is proportional to the total new information:


(3.1) $$I_{\text{tot}}\sim B\sim N^{a}\left[1-{\left(1-p\right)}^{cN^{b}}\right].$$

Equation (3.1) predicts three regimes. First, there is a regime in which each additional organizational unit contributes more or less independently to the expertise of the firm, maximizing the benefit of each organizational addition. Then, there is a relatively sharp, exponential turning point at which the organizational units suddenly saturate the space of information and when every new piece of information almost always intersects with some unit’s expertise. Thereafter, benefits from filling the space are marginal, but the rate of new information is only determined by a universal rate $N^{a}$ at which organizations read. Since this is also the regime in which organizational units overlap in expertise, this is compatible with the observation that for large firms, the dominant limit is not cumulative expertise but the fact that units must coordinate. Thus, equation (3.1) presents a testable prediction for how the way that firms fill the space of information and the onset of a coordination problem manifests in the cost of reading.

To fit the model to the data, we minimize the least-squares error on a logarithmic scale. While we have a sufficiently wide range of data to simply fit articles and sources, we are limited in the number of topics, which leads the curve to flatline at about $R\sim 10^{3}$ . A natural solution seems to be to fit to the part of the curve that comes before the flatline, but this leads to the problem of choosing the maximum values $R_{\text{max}}$ below which to fit. Since there is no definitive point to which to restrict ourselves, we instead vary the cut-off $R_{\text{cut-off}}$ over a wide range. Helpfully, we find the errors to be large when we restrict ourselves to fit a range $10^{2}≲R≲10^{3}$ records, but they suddenly drop at larger R . There, we find a range in which the exponent a that determines the extrapolated region stays within a narrow range. Finally, we find beyond $R≳10^{3}$ or $R≳10^{4}$ (depending on the lower cut-off) that the exponent for the mean curve a exceeds that for the maximum curve, which is physically impossible and indicates that the curves overfit the flatline (see appendix D for more details). Thus, we find a natural fitting regime in which the extrapolation remains consistent while not overfitting the data cut-off.

Remarkably, our model matches closely the curves in figure 3. Using the scaling relationship between records R and employees N to replace N with R , we fit the prediction in equation (3.1) to the Heaps’ plots in figure 3. The mean and maximum curves, shown in the blue and orange curves respectively, agree extraordinarily well with the data. Importantly, our estimate for the exponent b , how team size scales with the number of employees, are all sublinear. For the mean curves, we find for articles, sources and topics, b≈3/10 , b≈1/3 and b≈1/2 , respectively. For the maximum curves, we find b≈3/10 , b≈2/5 and b≈2/3 . Sensibly, the increasing values indicate that bigger collections of employees map onto larger aggregates of information.

The observation that larger groups matter for larger aggregations of information also manifests at the values of R at which the maximum variety curves peel away from the one-to-one line. When the inflection point is defined as the number of records at which the quantity in question first reaches 90% of linear growth, it occurs at R=1645 records for articles, R=41 records for sources and R=18 records for topics. Using our measured scaling relationships, the points correspond to publicly listed assets and annual sales of typically $440 million & $44 million, $30 million & $2 million and $16 million & $1 million, respectively. That the variation of the inflection point maps to firms of different sizes suggests that firms may pass through critical sizes at which the amount of new information of a certain granularity cannot be processed in the same way.

## 4. Information intensive versus extensive growth

As the final comparison, we consider how firm size relates to topic variety. As a start, we might anticipate three qualitatively different scenarios relating size such as assets A with topics read T , or the relationship $A\sim T^{\gamma }$ for positive exponent γ . If firms were to focus on core competencies, we might expect superlinear scaling of γ>1 since that means firms tend to reinvest topics that they already read. Then, we would expect that the ratio of assets per topic $A/T\sim T^{\gamma -1}$ increases with *T *. In the linear case γ=1 , we would find that a fixed unit of asset growth corresponds to the addition of a topic, or a siloed portrait of a firms such as might be expected from the separate acquisitions of a conglomerate that remain unintegrated. The final, sublinear case γ<1 would be unusual because it would suggest that assets are being divested from existing interests and spread more thinly across an enlargened set. Since larger firms tend to be older [32], the ratio of A/T may reflect an aspect of strategy that we distinguish as either ‘intensive’ (superlinear) or ‘extensive’ (sublinear).

To determine γ , we must rely on the previous relationship for assets A as a function of records R from figure 1, which was approximately $A\sim R^{3/4}$ , and the predicted relationship between topics T and R given by exponent a measured in figure 3. For firms of mean reading variety, we found median exponent values a≈1/4 and for firms of maximal variety we found a≈1/2 . Putting the relationships together, we estimate for large firms the relationships $A\sim T^{3}$ and $A\sim T^{3/2}$ , respectively. Confidence intervals of 95% on the values of a range from [0.05,0.34] to [0.28,0.78] correspond to γ in the intervals [2,14] and [0.9,2.5] , respectively; thus, the relationship between A and T remains mostly confined to the superlinear regime. Overall, this is consistent with the picture of a firm that retains a fixed set of interests into which it reinvests assets, although the error bars also allow for instances of alternative ‘silo’ and extensive strategies. Taken together with our other findings, this observation indicates how the information dimension may reflect elements of organizational structure.

## 5. Discussion

How groups of biological organisms exchange information to coordinate individual components has been a major area of interest in the study of collective behaviour [3,5,35]. Yet, it is difficult to query the cognitive state of the individual, and a sophisticated experimental apparatus is crucial to control and track the information that individuals are receiving and generating [3,36–39]. In organizations, much transmitted information is recorded and its content understandable; instead, the scale and complexity of the information is little understood quantitatively. Thus, the firm presents a complementary opportunity to study information flows. We use such information at a wide scale to study the amount of information that organizations, primarily firms, consume and focus on how such information is connected to firm size.

We first show a sublinear relationship between the size and the information footprint of a firm in figures 1 and 2. The main implication of the sublinear scaling is that the effective cost of an additional unit of information shrinks, signalling an ‘economy of scale’. This observation aligns with the intuition that the tools of the knowledge economy such as information technology enhance firm productivity [40,41]. As part of this, the typical large firm accesses more *records* and consumes more *articles* per employee, which implies that its employees on the whole are more productive at processing elementary information. The sublinear scaling leads to an information consumption inequality that is an exaggerated version of the economic inequality between firms given by the classic finding of Zipf’s law in US Census data [29]. We validate this by measuring the exponents of power-law tails in the distributions of firm information consumption α , finding an exaggerated tail for records and articles of α<2 . Sources, in contrast, show nearly linear scaling, and the power law tail is correspondingly Zipfian, or α=2 . Flipping the axes around by taking information as the driver, the inverse perspective is of a superlinear increase in information required to ‘power’ unit economic growth, or one of diminishing returns. The two perspectives, while compatible with the same scaling laws, correspond to different causal mechanisms for the drivers of productivity in firms.

The sublinear and linear scaling depending on information granularity is curious because the patterns are at odds with the classical idea behind labour specialization. As employees become more specialized at their jobs, specialization should trim overhead, implying that information overhead should also decrease. Then, labour specialization implies decreased reading per employee, reduced exploration and superlinear scaling. Neither the scaling patterns for records and articles substantiate this hypothesis nor do the aggregate quantities of reading such as sources and topics for which the scaling with economic measures is close to linear as is summarized in the scaling exponents β in figure 2. As we show in the electronic supplementary material, table S2, we find linear and sublinear scaling on all counts, indicating an unexplained aspect of labour specialization in terms of reading that deserves further study.

One possible reason for the change from sublinear to linear scaling with information granularity is that processing diverse information at larger scales is more complicated. Gaining something out of a whole new topic may require restructuring such as adding a division or changing the corporate mission, whereas reading a new article on the same topic is trivial. This would mean that firm growth in information space at the coarsest levels is more tied to economic growth, echoing the role of intangible aspects in determining firm costs [42]. The observations suggest two different ways in which information costs may reflect firm growth, either through an increasing economy of scale or reflecting the demands of diversification.

As a second observation, we show that the volume of firm online reading is limited in its variety because the number of articles, sources and topics scales sublinearly with reading volume in the Heaps’ plots of figure 3. Even the most diversely read firms are redundant readers above a critical size, and the critical size depends on information granularity. By considering how the expertise of organizational units tile the space of information content (figure 4), we predict the emergence of a transition when the organizational units begin to overlap in expertise. Overlap means the point at which coordination or conflict between units becomes an issue. This model yields a prediction that fits the data remarkably well, indicating that organizational constraints may leave traces in the information footprint.

**Figure 5.**
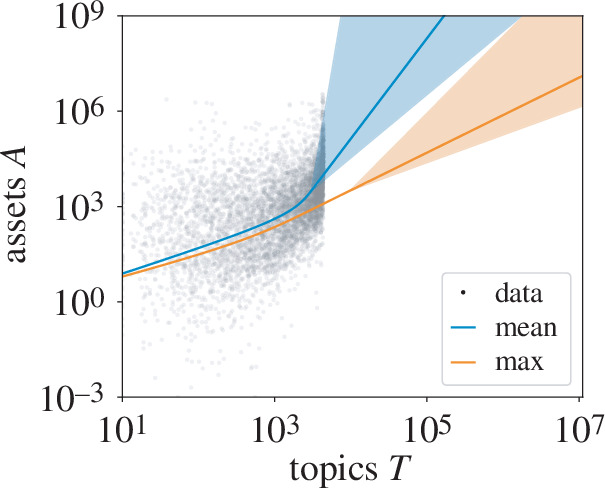
Predicted relationship between assets and topics from combining the information processing model from figure 3 and the scaling between assets and records (see the electronic supplementary material, table S2). Grey points indicate public firms. A superlinear curve in the tail indicates increasing assets per topic read, whereas a sublinear curve decreasing assets per topic read, or an intensive versus extensive strategy, respectively. Linear scaling indicates proportional growth in assets with topic variety such as if investments were split equally across them. Best estimates for scaling in the tail are approximately $A\sim T^{3}$ for the mean and $A\sim T^{3/2}$ for the max, which both correspond to intensive strategies. Shaded regions indicate 95% confidence intervals as defined in electronic supplementary material, appendix D. Bottommost error bars extend to sublinear scaling, or roughly as $A\sim T^{9/10}$ .

Finally, we connect reading variety to firm size by using the scaling relationships found in the previous analyses (figure 5). Inspired by the qualitative differences that could emerge for the scaling relationships relating the two properties, we predict three types of firm strategy that would correspond to each one. The differences are summarized in the ratio of assets to topics A/T that either grows, stays constant or shrinks with firm size. We find that nearly the entire range compatible with the fit to data corresponds to an increasing ratio, or an intensive strategy, consistent with other measures [43]. This is in contrast with the economy of scale for records and articles. The difference may reflect the fact that expanding the interests of a firm is not as simple as reading another article, but it may involve a structural cost (e.g. hiring the right employees and setting up management structures) reflected in processing higher levels of information.

Deviations from the trends indicate sector or firm-specific behaviours that may impact performance and explain non-financial outcomes. As an example of this, we consider deviations from the scaling fits in figures 1 and 2, representing either firms that show excess reading (negative logarithmic errors) or under reading (positive logarithmic errors). Sensibly, we find that excess reading is correlated with operational diversity, segment sales diversity (defined as the Herfindahl–Hirschman index, a measure of market concentration of sales across Standard Industrial Classification codes in 2019) and intellectual property diversity (defined as the patents across patent classes for patents granted after 2010 to 2022).^2^ For all measures, the relationship between excess reading and intellectual property (IP) diversity is strong.^3^ Notably, excess reading is robustly correlated with higher future returns from 2019 onwards and higher valuations in 2019 across assets, PPE and employees. A measure of future earnings potential, Tobin’s *Q*, is strongly and robustly correlated with excess reading (table 1). The pattern holds when we control industry fixed-effects and is emphatically stronger than compared to measures of firm size. The magnitude of the effects is sizeable. For example, a s.d. change in deviation excess reading corresponds to roughly 20% increase in Tobin’s *Q* and a 11% increase in returns the following year. While the various measures of diversity also increase with economic size, subtracting off the baseline trend with the scaling model is a much stronger indicator of future valuation (see the electronic supplementary material, tables S3–S13). Thus, deviations from cross-industry trends in terms of information consumption—differently from raw measures of economic size—is robustly correlated with several measures of financial performance.

**Table 1.** Regression table for measures of firm performance and excess reading. (The analyses include winsorization at the 1% level for all continuous variables. Diversity is measured in 2018 but valuations and returns are for 2019. The coefficients are normalized by standard deviation (i.e. a standard deviation increase in excess reading leads to a 20% increase in Tobin’s Q relative to the standard deviation in column 6) but not for returns per standard description in finance. Standard errors, reported in parentheses, are clustered by firm. ***, ** and * denote statistical significance at the 1%, 5% and 10% confidence levels, respectively.)

	IP diversity		sales diversity		Tobin *Q*		return	
	(**1**)	(**2**)	(**3**)	(**4**)	(**5**)	(**6**)	(**7**)	(**8**)
constant	−1.437***		−0.5773***		0.0005		−0.4395***	
	(0.1555)		(0.0933)		(0.0837)		(0.1196)	
excess reading (assets)	0.2344***	0.2461***	0.1077***	0.0453*	0.2926***	0.2020***	0.1970***	0.1085**
	(0.0560)	(0.0541)	(0.0238)	(0.0268)	(0.0231)	(0.0263)	(0.0369)	(0.0424)
log assets	0.1913***	0.2275***	0.0761***	0.0941***	−0.0037	−0.0017	0.1491***	0.1248***
	(0.0191)	(0.0190)	(0.0125)	(0.0118)	(0.0111)	(0.0111)	(0.0161)	(0.0167)
observations	967	967	3149	3149	3149	3149	3187	3187
*R* ${,}^{2}$	0.10518	0.21661	0.01514	0.09205	0.09974	0.15819	0.02669	0.06808
within *R* ${,}^{2}$		0.14613		0.03391		0.03870		0.02147
NAICS2 fixed effects		✓		✓		✓		✓

Zooming out to the macroscopic trends, we find indications that organizational units, not the individual employees, play a role in information consumption. The observations echo the classic ideas that organizational structures are crucial for the ‘absorptive capacity’ of a firm [13], the way knowledge is stored or exploited [44], the role of teams [45] and performance [46]. Here, we provide another piece of the puzzle by measuring aspects of information consumption that elucidate the information behaviour of employees. Information use by individuals follows a parallel line of work in biological collectives, where we have begun to connect individual-level information exposure and cognition to group-level capabilities [47–50]. In the context of firms, it is still an open question how information consumption captures the capacity or interaction structure. As a start, variance in information use suggests one way to distinguish organizational structures and to highlight unusual ones. Large deviations from patterns extracted over many millions of firms are likely to represent surprising activity that demands further attention. In this sense, our work suggests a way forward for understanding why and how firms use information and the principles that organize information processing in collective behaviour.

## Data Availability

Reading data is commercial property that comes from a data partner (as further detailed in the electronic supplementary material) [51]. The data are proprietary and neither the data nor the identity of the data partner can be disclosed due to binding legal agreements. Code excluding explicit references to the original data source is available at [52].

## Footnotes

^1^
If it were to pay more than for itself, we would anticipate that more information would be consumed, and if it paid for less, then the amount would be reduced. In other words, the marginal benefit should equal the marginal cost.

^2^
The long sample accounts for the infrequency of patents. If there are no patents, the firm is excluded from the sample.

^3^
Sales diversity holds only for excess reading with respect to assets and PPE but not with sales and annual employees; this may reflect noise in the sales diversity measures, which are optionally reported by companies.

## Authors’ notes

Advice was sought from our data partner’s legal team regarding potential hazard in identifying data sources in the published work. On the advice of the legal team of our data partner, the published version of the paper has had the names of the data providers redacted. Readers should contact the authors of the paper for further details.
